# Loneliness and marijuana use among Older Adults: An examination of the 2018 Health and Retirement Study

**DOI:** 10.1093/geroni/igab046.2527

**Published:** 2021-12-17

**Authors:** Jie Yang, Andrew Yockey

**Affiliations:** 1 East Carolina University, Greenville, North Carolina, United States; 2 University of North Texas Health Science Center, Denton, Texas, United States

## Abstract

Introduction: There is substantial literature to suggest that loneliness is a risk factor for marijuana initiation, use, and continued use into adulthood. However, these relationships have yet to be investigated among older adults. Given that recent research suggests marijuana use is increasing among older adults, the purpose of the present study was to examine loneliness and other risk factors among a national sample of older adults ages 50 years or older. Methods: A secondary data analysis was conducted on the 2018 Health and Retirement Study (HRS) was conducted (n = 1,431). The HRS is a national, biannual survey conducted in the United States to assess health, psychosocial, and demographic questions among adults ages 50 years or older. We created a loneliness scale from the available questions and assessed differences based on demographics, lifetime use, and past-year use of marijuana. Weighted analyses with cyclical tree-based hot-deck imputation were conducted. Results: A sizeable percentage (23.5%) of older adults have ever used marijuana and a considerable amount (14.8%) of adults have used marijuana in the past year. Differences were found based on sex (p <.0001), age (p <.0001), race (p <.0001), and income (p <.0001). Loneliness significantly predicted marijuana usage, with adults who reported loneliness nearly 5 times more likely to use marijuana (aOR: 4.87, 95% CI 3.89, 6.10). Discussion: The present study investigated loneliness and marijuana usage among a national sample of adults. Findings from the present study may inform behavioral health interventions, harm reduction, and gerontological health.

